# Neurocysticercosis presenting as a locked-in lateral ventricle: A case report and evidence-based review

**DOI:** 10.1016/j.idcr.2023.e01778

**Published:** 2023-04-27

**Authors:** Moustafa A. Mansour, Mohamed Tahir, Zarina Ahmadi

**Affiliations:** a Department of Neurology and Neurologic Surgery, Faculty of Medicine, Al-Azhar University, Cairo, Egypt; b Department of Neurology and Neurologic Surgery, Mayo Clinic, Rochester, MN, USA; c Division of Neuro-Intensive Care, Dar Al-Fouad Medical Corporation, Cairo, Egypt; d Department of Emergency Medicine and Critical Care, Faculty of Medicine, Al-Azhar University, Cairo, Egypt; e Department of Diagnostic and Interventional Radiology, Faculty of Medicine, Al-Azhar University, Cairo, Egypt; f Department of Infectious Diseases and Tropical Medicine, Faculty of Medicine, Al-Azhar University, Cairo, Egypt; g Department of Internal Medicine, Faculty of Medicine, Al-Azhar University, Cairo, Egypt

**Keywords:** Neurocysticercosis, Hydrocephalus, Locked-in ventricle, CNS infections, Parasitic infestation, Case report

## Abstract

Human neurocysticercosis is one of the most prevalent parasitic infestations of the central nervous system. It is considered the most frequent underlying etiology of acquired epilepsy in endemic areas in Central and South America, East Europe, Africa, and Asia, with over 50 million people affected globally. Ventricular involvement is a severe form of neurocysticercosis commonly manifests as arachnoiditis, raised intracranial pressure, or hydrocephalus, secondary to CSF flow obstruction of the ventricular system by cysts of *Taenia solium*, hence requiring prompt, aggressive intervention to alleviate the increased intracranial pressure to prevent imminent lethal complications. Ventricular neurocysticercosis can involve any brain ventricle but with a paramount preference for the fourth ventricle, causing non-communicating hydrocephalus and symmetric ventriculomegaly. However, in this clinical report, we present an uncommon case of trapped (locked-in) lateral ventricle caused by an isolated cysticercus trapped at the ipsilateral foramen of Monro, which is an atypical location for neurocysticercosis, adding more challenges to diagnosis and during the process of surgical extraction. We additionally provide a comprehensive, evidence-based review of the clinical course and management options relevant to the entity of ventricular neurocysticercosis, besides recent relevant clinical updates.

## Highlights

• Neurocysticercosis (NCC) is the most prevalent CNS parasitic infestation globally.
• Ventricular NCC (vNCC) typically presents as hydrocephalus.
• An isolated (locked-in) ventricle is an uncommon presentation of vNCC.
• 3D CISS MR imaging is the most sensitive and specific modality in detecting vNCC.
• Endoscope-assisted extraction is now the most widely preferred method for vNCC.

## Introduction

As the name suggests, cysticercosis is a parasitic infestation caused by the larval stage of *Taenia solium* tapeworm (cestode), which develops after ingesting eggs from the feces of a tapeworm carrier (fecal-oral transmission) or eating meat from an infected intermediate host [1]. Cysticercosis has a higher tendency toward skeletal muscles, brain, and spinal cord than any other body organ, considered the most common cause of neurologic parasitic infestations [2]. Cysticercosis is highly endemic in developing countries due to poor socioeconomic and sanitation conditions [3], with neurocysticercosis being set as the most common etiology of acquired seizures in these regions [4]. Neurocysticercosis-induced seizures are most commonly observed in cases with brain parenchymal involvement. However, the ventricular form of neurocysticercosis tends to present differently [5]. Clinical presentation of the ventricular form of neurocysticercosis is primarily due to CSF flow obstruction, which can be complete, partial, or transient obstruction [6], [7], [8]. Furthermore, degenerating cysticerci might get adherent to the ventricular ependymal lining, causing acute or chronic life-threatening ventriculitis [9], [10]. Ventricular neurocysticercosis most commonly presents with signs and symptoms of hydrocephalus, periventricular inflammation, and in severe cases, a locked-in ventricle with imminent herniation if untreated promptly [11], [12]. Therefore, ventricular neurocysticercosis is considered a critical entity, requiring prompt, aggressive interventions in view of its rapidly progressive clinical course and potentially lethal complications.

## Case presentation

**Fig. 1:**
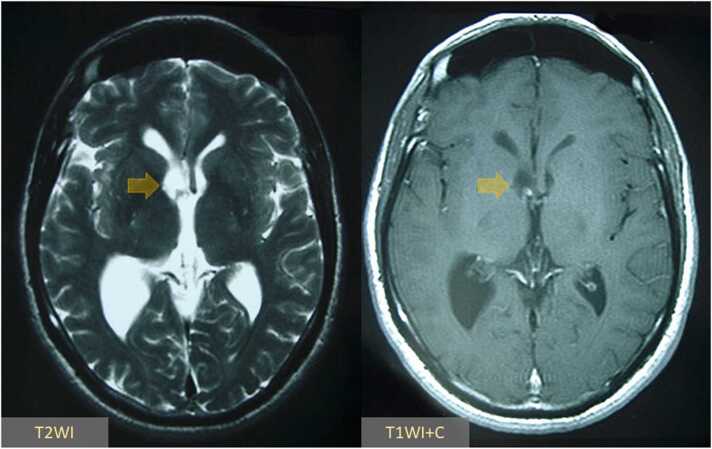
Axial T2WI and postcontrast T1WI at the level of the lateral ventricles demonstrate a well-delineated, thin-walled, non-enhancing cystic lesion with an eccentric mural nodule in the right-sided lateral ventricle (arrows), causing outflow obstruction through the ipsilateral interventricular foramen of Monro.

A 42-year-old man presented to the emergency department after waking up with a severe generalized headache and profuse vomiting. He reported a 2-week history of progressive headaches and blurry vision, partially controlled with OTC analgesics. On neurologic examination, he had preserved motor, sensory, and autonomic functions with unremarkable neurologic reflexes throughout. However, his clinical manifestations were suggestive of increased intracranial pressure (ICP); therefore, it was prudent to proceed with head imaging before any laboratory investigations or further clinical examinations. A CT scan of the head demonstrated an expanded lateral ventricle on the right side without any significant mass effect, consistent with an isolated, right-sided non-communicating hydrocephalus. However, no identifiable cause was evident. Therefore, we scheduled the patient for urgent MR imaging, which revealed a well-delineated, thin-walled, non-enhancing cystic lesion with an eccentric mural nodule in the right-sided lateral ventricle, causing outflow obstruction through the ipsilateral interventricular foramen of Monro (Fig. 1). Analysis of blood and CSF samples revealed normal ranges, except for mild hyperproteinorrachia (109 mg/dl) and lymphocytic pleocytosis (95%). CSF bacterioscopy was negative, but ELISA testing demonstrated positivity for cysticercus antibodies.

**Fig. 2:**
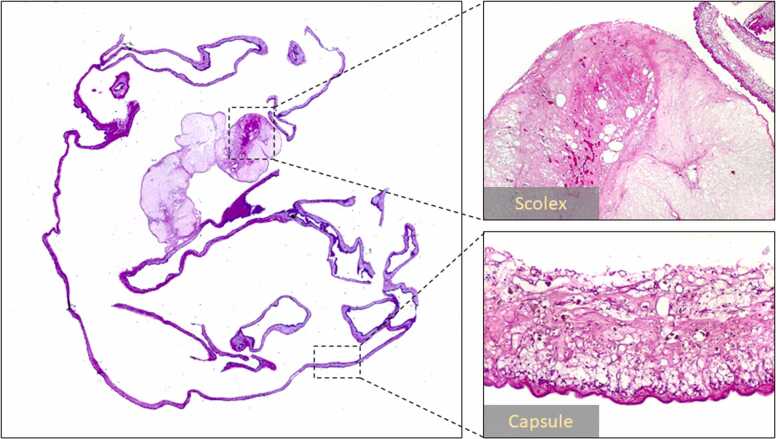
Histopathological images of the excised lesion demonstrate a nonviable, necrotic scolex projecting from the interior surface of a viable cystic membrane that is externally covered by numerous sets of microvilli.

Given the data obtained thus far, we made a preliminary diagnosis of neurocysticercosis-induced hydrocephalus. The patient was then taken to the OR, where an endoscopic transventricular approach was performed to extract the cystic lesion. Concurrently, an external ventricular drain (EVD) was placed on the ipsilateral side, which instantly alleviated the high ICP manifestations. The cyst was subsequently transferred to the histopathology department. Upon opening the cyst by cutting its membrane, an elongated whitish structure, in continuity with a certain point on the inner surface of the membrane, was observed, consistent with the scolex (larval head) of the tapeworm. Histopathological studies on the excised cysticercus demonstrated a nonviable, almost necrotic, vacuolated scolex with a still-viable cystic membrane (Fig. 2). Based on the obtained histopathological results, we were able to confirm our preliminary diagnosis of neurocysticercosis, to start the patient on high-dose dexamethasone followed by albendazole to eliminate any still-unidentifiable minute cysticerci. Spinal MR imaging was conducted to screen for any associated spinal cysticerci as per the institution's guidelines, but the results were unremarkable. Three days after the procedure, the EVD was safely removed, and the patient was able to resume his daily activities. Two months after being discharged, he showed up for a follow-up as per our recommendation. Follow-up MR imaging demonstrated normal-sized ventricles, and the CSF ELISA testing exhibited negative results.

## Discussion

**Fig. 3:**
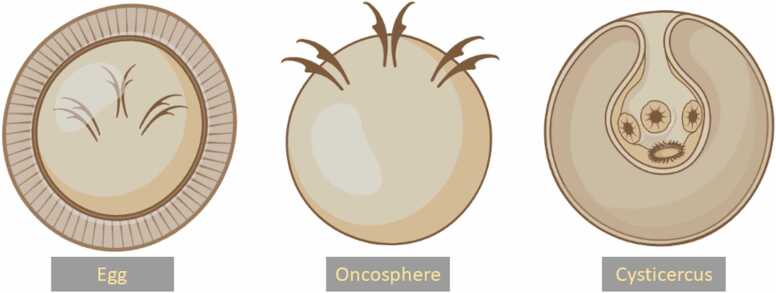
A schematic illustration of *Taenia Solium* egg, oncosphere, and cysticercus.

Humans are considered definite hosts for *T. solium* after ingesting raw or undercooked meat-containing cysticerci from infected pigs, which act as intermediate hosts. However, humans might act as intermediate hosts if the infection has occurred through the ingestion of eggs rather than cysticerci via a fecal-oral route. Within the intermediate host, the egg hatches and transforms into an oncosphere, which is the larval form of *T. solium* (Fig. 3). After burrowing through the host's intestinal wall, the oncosphere gains access to blood circulation, eventually developing into a cysticercus or bladderworm inside the CNS [13]. Neurocysticercosis can be divided into intracranial and extracranial (i.e., spinal) forms, with the latter only accounting for 0.7–11.1% of all neurocysticercosis cases [14], [15]. The intracranial forms can be further classified into parenchymal, ventricular, and cisternal (subarachnoid) subtypes based on the affected brain area. Ocular involvement may, however, be included as a fourth intracranial subtype in some classifications. Brain parenchyma is the most affected area, while the involvement of the subarachnoid space is the least common (∼12%) but the most lethal form of intracranial neurocysticercosis [16]. The most frequent brain parenchymal location is in the cerebral hemispheres, typically at the gray-white matter junctions. Cerebellar involvement, on the other hand, is rare, with only a few reports available [17], [18], the issue that might be due to the poor cerebellar vascular circulation compared to cerebral circulation.

Ventricular involvement accounts for approximately 20% of all intracranial neurocysticercosis. Oncospheres, by the general circulation, reach the ventricles via the choroid plexus, where they transform into cysticerci. Apuzzo *et al.* reported through their case series that included 45 patients with intraventricular neurocysticercosis that the fourth ventricle was the most affected brain ventricle (∼53%), given its most dependent location, followed by the third ventricle (∼27%). The lateral ventricle and Sylvian aqueduct accounted for only 11% and 9%, respectively [6]. Ventricular neurocysticercosis can present as isolated cysts, ependymitis (ventriculitis), or an amalgamation of both, leading to CSF outflow obstruction and ventriculomegaly. Depending on parasite burden, cyst location, parasite degradation, and host inflammatory response, intraventricular neurocysticercosis can present with a wide spectrum of clinical manifestations. However, most patients typically present with raised ICP-related manifestations due to non-communicating hydrocephalus [19]. Given the location where cysticerci reside, patients with isolated lateral ventricular involvement typically exhibit high intracranial pressure with a resultant mass effect, compressing the neighboring structures, as perfectly demonstrated in our case [20], [21], while patients with third ventricular involvement can present with more serious manifestations such as suddenly disturbed consciousness level and visual disturbance due to the mass effect exerted on the diencephalon, which hypothetically might lead to sudden death as seen with colloid cysts located in the same area. Cysticerci occupying the fourth ventricle usually present with subacute hydrocephalic manifestations with/without signs of brainstem affection [7]. Moreover, fourth ventricular neurocysticercosis might also present as Bruns’ syndrome, which is a condition characterized by episodic events of high intracranial tension induced by rotatory head movements of the head, followed by rapid recovery [22].

Brain imaging plays a pivotal role in neurocysticercosis diagnosis. Due to the readily availability of CT scans in most healthcare centers, it is widely used as the initial diagnostic modality. CT scans have shown higher sensitivity and specificity for parenchymal neurocysticercosis, compared to ventricular lesions, which are hard to visualize via brain CT because they appear as thin-walled lesions, exhibiting isodense signal changes similar to the CSF [23], [24]. Ventricular neurocysticercosis typically presents on CT scans as a resultant ventricular expansion with/without exhibited hydrocephalus, depending on their number and location [25]. However, direct visualization of the cysts using this radiological modality is not typically feasible, requiring a more sensitive modality such as MRI or the less commonly used invasive direct contrast ventriculography [25]. On MRI, intraventricular cysticerci typically demonstrate signal intensity values similar to the CSF with a well-delineated, thin-walled cystic membrane [26]. Although these lesions exhibit a hyperintense signal on T2WI, similar to CSF, they sometimes might demonstrate lower intensities than CSF due to their higher protein content [27]. Conventional MRI modalities can indeed provide better visualization than CT scans; however, they still may miss some intraventricular cysticerci [28]. The most optimal MRI protocol for the identification of intraventricular cysticerci is still controversial. However, recent data suggests that the 3D constructive interference in steady-state (CISS) MRI modality is more sensitive and specific than routine MRI sequences in detecting intraventricular cysticerci [28]. Additionally, some available reports demonstrated that obtaining 3D spoiled gradient recalled echo MRI sequences can help better the detection of intraventricular cysticerci [29]. Furthermore, diffusion-weighted MRI has been shown to be very specific, especially in cysticerci harboring scoleces, as these scoleces typically demonstrate diffusion restriction signal changes [30]. Other advanced imaging modalities such as perfusion and spectroscopy are nonspecific but help to differentiate neurocysticercosis from other potential diagnoses such as brain tumors. Pandit *et al.* reported that a combination of elevated lactate, choline, alanine, and succinate levels, on top of suppressed creatine and NAA levels to be characteristic of parenchymal lesions [31]; however, no available reports regarding whether the intraventricular cysts will exhibit the same findings or will possess unique spectroscopic findings. Other authors have shown that using FLAIR MRI after five minutes of 100% oxygen inhalation can improve the detection of subarachnoid and cisternal cysticerci by increasing the CSF signal intensity, leading to a greater conspicuity of cyst walls [32]. However, this method cannot be applied to cysts involving the ventricular spaces because the CSF radiological signal inside the ventricles is unlikely to change in response to 100% oxygen inhalation.

Several serological tests are currently available to diagnose neurocysticercosis by detecting antibodies to *T. solium*-related antigens in serum and CSF. However, available data demonstrate that standard enzyme-linked immunosorbent assays are neither sensitive nor specific for neurocysticercosis diagnosis [33]. The enzyme-linked immunoelectrotransfer blot (EITB) is considered the current test of choice for cysticercosis, with a 96% sensitivity and almost 100% specificity [34], [35]. However, the sensitivity might be lower in patients harboring few cysts or whose cysts are calcified or degenerated [36]. In a recent study by Arora *et al.*, the authors demonstrated that the presence of a 15 kDa reactive band on EITB is highly sensitive and specific for the diagnosis of neurocysticercosis, although the presence of a 35 kDa band might be associated with infection by multiple cysticercus lesions [37].

Neurocysticercosis can be treated medically with cysticidal agents and corticosteroids or surgically to remove the cyst(s). Praziquantel and albendazole are the commonly used cysticidal drugs that have demonstrated high efficacy against all forms of neurocysticercosis. However, they should be preceded by steroid administration, especially in patients with the ventricular and subarachnoid subtypes, to reduce the resultant inflammation from the parasitic degradation and the subsequent risk of shunt obstruction [38]. In a clinical trial by Göngora-Rivera *et al.*, the authors reported that albendazole at 30 mg/kg/day plus dexamethasone for eight days was both safe and effective in the clearance of intraventricular and subarachnoid cysts [39]. In the treatment of intraventricular and subarachnoid cysts complicated with hydrocephalus, a ventriculoperitoneal (VP) shunt is warranted as early as possible to alleviate the high intracranial pressure and reduce the associated morbidity and mortality. A VP shunt is also warranted if ependymitis is detected to prevent imminent hydrocephalus that might be life-threatening. Shunt failure in cases with the ventricular subtype of neurocysticercosis rates can be as high as 30–70%. Therefore, some patients might require multiple revisions of the inserted shunts. Removal of the ventricular cysts can be performed via several approaches, ranging from closed aspiration to an open craniotomy. The transventricular endoscopic approach is now the most widely preferred method due to the associated lower rates of morbidities and mortalities. However, potential limitations of the neuro-endoscopic approach do exist, including intraventricular hemorrhage. Furthermore, proceeding with the endoscopic approach in the presence of ependymitis might be hazardous [40]. Aspiration of the cyst contents before its extraction to shrink it was found to make the cyst easier to extract, while intra-operative rupture or incomplete removal of the cyst has no negative sequel on the outcome [41].

Neurocysticercosis generally possesses a favorable general prognosis after initiating treatment. However, parenchymal involvement might lead to epilepsy and recurrent seizures, while subarachnoid and ventricular subtypes might lead to permanent hydrocephalus, intracranial nerve entrapment leading to cranial nerves palsy, or cerebrovascular complications such as ischemic infarction, transient ischemic attacks, and brain hemorrhages [42]. Therefore, early detection and prompt treatment are the mainstays in preventing these lethal neurocysticercosis-associated complications and improving long-term outcomes.

## Take-home points

• Neurocysticercosis is the most prevalent parasitic infestation of the CNS, with approximately 50 million people affected globally.
• It develops after ingesting eggs from the feces of a tapeworm carrier (fecal-oral transmission) or eating meat from an infected intermediate host.
• Seizures are the most commonly observed complication in cases with brain parenchymal involvement.
• Hydrocephalus, symmetric ventriculomegaly, and increased ICP are the most common presentation of ventricular neurocysticercosis, while an isolated ventricle is uncommon.
• The 3D constructive interference in steady-state MR imaging modality is more sensitive and specific than routine MRI sequences in detecting intraventricular cysticerci.
• FLAIR MR imaging modality, preceded by a five-minute 100% oxygen inhalation, provides a feasible, inexpensive method for detecting cisternal and subarachnoid cysticerci; however, further modifications are still required to enable the visualization of the intraventricular cysts.
• EITB is considered the current serological test of choice for neurocysticercosis.
• A VP shunt is warranted in ventricular neurocysticercosis if associated with increased ICP or if ependymitis is suspected.
• Transventricular endoscopic extraction is now the most widely preferred method for ventricular neurocysticercosis.
• Cerebrovascular complications of neurocysticercosis are more common than expected and typically associated with the subarachnoid and cisternal subtypes.

## Ethics approval

The ethics committee at Al-Azhar University hospitals officially approved all the performed procedures and the followed guidelines under the reference number of HSZ-22–001446.

## Contribution

**M.M.** was responsible for the conception of the work, data collection, drafting the article, critical revisions, and obtaining approval of the final version of the manuscript. **M.T.** and **Z.A.** contributed by drafting the article, and critical revisions. All authors were involved in direct patient care.

## Consent to participate

The patient provided a written consent to publish the case and related data.

## Funding

None declared.

## Declaration of competing interests

None.
